# Molecular epidemiological investigation of group A porcine rotavirus in East China

**DOI:** 10.3389/fvets.2023.1138419

**Published:** 2023-03-21

**Authors:** Ran Tao, Xinjian Chang, Jinzhu Zhou, Xuejiao Zhu, Shanshan Yang, Kemang Li, Laqiang Gu, Xuehan Zhang, Bin Li

**Affiliations:** ^1^Institute of Veterinary Medicine, Jiangsu Academy of Agricultural Sciences, Key Laboratory of Veterinary Biological Engineering and Technology, Ministry of Agriculture, Nanjing, China; ^2^Jiangsu Key Laboratory for Food Quality and Safety-State Key Laboratory Cultivation Base of Ministry of Science and Technology, Nanjing, China; ^3^Jiangsu Co-innovation Center for Prevention and Control of Important Animal Infectious Diseases and Zoonoses, Yangzhou University, Yangzhou, China; ^4^College of Veterinary Medicine, Hebei Agricultural University, Baoding, China

**Keywords:** porcine rotavirus, prevalence, East China, VP7, VP4

## Abstract

Group A porcine rotavirus (RVA) is a serious threat to the breeding industry worldwide, which was associated with severe diarrhea in piglets. However, the prevalence and molecular characterizations of RVA circulating in farms of East China remains largely unknown. Five hundred and ninety-four samples were collected from 35 farms in East China from September 2017 to December 2019. The results showed that 16.8% was positive for RVA of all samples. Among different types of samples, the highest positive rate of RVA was intestinal samples (19.5%), and among pigs at different growth stages, the highest detection rate of RVA in piglets was 18.5%. Furthermore, the VP7 and VP4 genes of nine positive samples were sequenced for alignment and phylogenetic analysis. Phylogenetic analysis revealed that the nine isolates belong to four kinds of genotype combinations correspondingly: G9P[7](5/9), G5P[13](2/9), G9P[13](1/9), and G5P[7](1/9).The data suggested that multiple genotypes combinations of RVA were circulating in pigs in East China. Thus, it's necessary to continuously survey the prevalence of RVA in pigs, aiding the rational application of vaccines or other measures for the prevention and control of RVA spread.

## 1. Introduction

Rotaviruses (RVs) are one of the main pathogens implicated in acute diarrhea in children and young animals, including nursing and weaned piglets (1–3). RVs mainly infect and disrupt small intestinal mature enterocytes and enteroendocrine cells leading to acute gastroenteritis, which are transmitted *via* the fecal-oral route (4). Porcine rotavirus (PoRV) was first isolated from infected pigs in 1976 (5). Subsequently, many studies documented the widespread prevalence of PoRV infections all over the world (3). PoRV had resulted in enormous economic loss in the worldwide swine industry since it was discovered (3, 6).

RVs are non-enveloped, segmented dsRNA viruses, which belong to the member of the family Reoviridae. Its genome was consisted of 11 segments encoding 6 structural proteins (VP1-VP4, VP6, and VP7) and 5 non-structural proteins (NSP1-NSP5/6). RVs are classified into 10 groups (A–J) based on the antigenic characteristics of VP6 gene (7). Besides, the G and P dual typing system was established, based on the outer capsid proteins VP7 and VP4 (8). To date, group A rotavirus (RVA), RVB, RVC, RVE, and RVH have been described in pigs (9, 10). However, among these groups of RVs, RVA represents the main cause of acute diarrhea in piglets because of its high prevalence and pathogenicity (11). Previous study showed that the prevalence rates of RVA in pigs vary from 3.3% to 67.3% (3). Furthermore, 12 G genotypes (G1-G6, G8-G12, and G26) and 18 P genotypes (P[1]-P[8], P[11], P[13], P[19], P[23], P[25], P[26], P[27], P[32], P[34], and P[49]) of RVA have been detected in pigs (11–20).

Until now, the surveillance of pigs RVA remains rare in China. Previous studies showed that the prevalence rate of porcine RVA was 28.76% in Shandong province and the dominant genotypes were G3, G5 and G9 (21). However, the prevalence genotypes and molecular characterization of RVA circulating in pigs in East China remains unknown. In this study, we investigated the prevalence and genetic characterization of porcine RVA in pig herds from East China (Jiangsu, Anhui, Shanghai, Zhejiang, and Shandong) from September 2017 to December 2019.

## 2. Materials and methods

### 2.1. Sample collection

From September 2017 to December 2019, a total of 594 samples (fecal swabs and small intestine tissue samples) were collected from 35 different pig farms in five provinces/cities (Jiangsu, Anhui, Shanghai, Zhejiang, and Shandong) in East China (details are summarized in Supplementary Table S1). Samples were collected from both healthy and sick pigs in different growing stages (within 30 days of piglets, 35–50 days of nursery pigs, 60–120 days of fattening pigs). There were no repeated samples collected from those farms. All collected samples were diluted in sterilized phosphate buffered saline (PBS), and centrifuged at 8,000 × g for 10 min. The supernatants were collected for further analysis.

### 2.2. RNA extraction and RT-PCR

Total RNA was extracted from pretreated samples using FastPure Cell/Tissue Total RNA Isolation Kit according to manufacturer's instructions (Vazyme, China). One-step RT-PCR was performed by using HiScript II 1st Strand cDNA Synthesis Kit (Vazyme, China) following the manufacturer's protocol. The partial VP6 gene and full-length VP7 and VP4 genes were amplified by using 2× Taq Master Mix (Vazyme, China). The primers sequences were presented in Supplementary Table S2. Briefly, a total of 12.5 μl 2× Taq Master Mix were mixed with the specific primer pair for the individual genes, 5 μl cDNA of each sample used as template, and nuclease-free water to 25 μl. The reaction conditions were as follows: initial denaturation at 95°C for 5 min, followed by 35 cycles of denaturation at 95°C for 30 s, annealing for 30 s, and extension at 72°C for 3 min, followed by extension fully at 72°C for 10 min. All RT-PCR products were analyzed on 1% agarose gels containing 0.5 μg/ml ethidium bromide and visualized under UV transilluminator.

### 2.3. VP7 and VP4 gene sequencing and phylogenetic analysis

Each PCR product was purified with the E.Z.N.A.^®^ Gel Extraction Kit according to manufacturer's instruction (Omega, China). The purified PCR products of VP7 and VP4 gene were cloned to pMD19-T vector (Takara, China), transformed to DH5α competent cells, and then cultured overnight at 37°C on Ampicillin agar plates. Recombinant DNA clones identified as positive for the respective gene by using PCR and then were sequenced by Sangon Biotech (Shanghai, China). The sequences were determined using Sanger sequencing and deposited in GenBank with accession numbers OP454313–OP454330, respectively (Supplementary Table S3). Sequences data were aligned with reference strains from GenBank using Clustal W and were analyzed using the software MEGA 11 (version 11.0.13) for phylogenetic analysis (the reference strains information was summarized in Supplementary Tables S4, S5).

## 3. Results

### 3.1. The prevalence of RVA in East China

**Figure 1:**
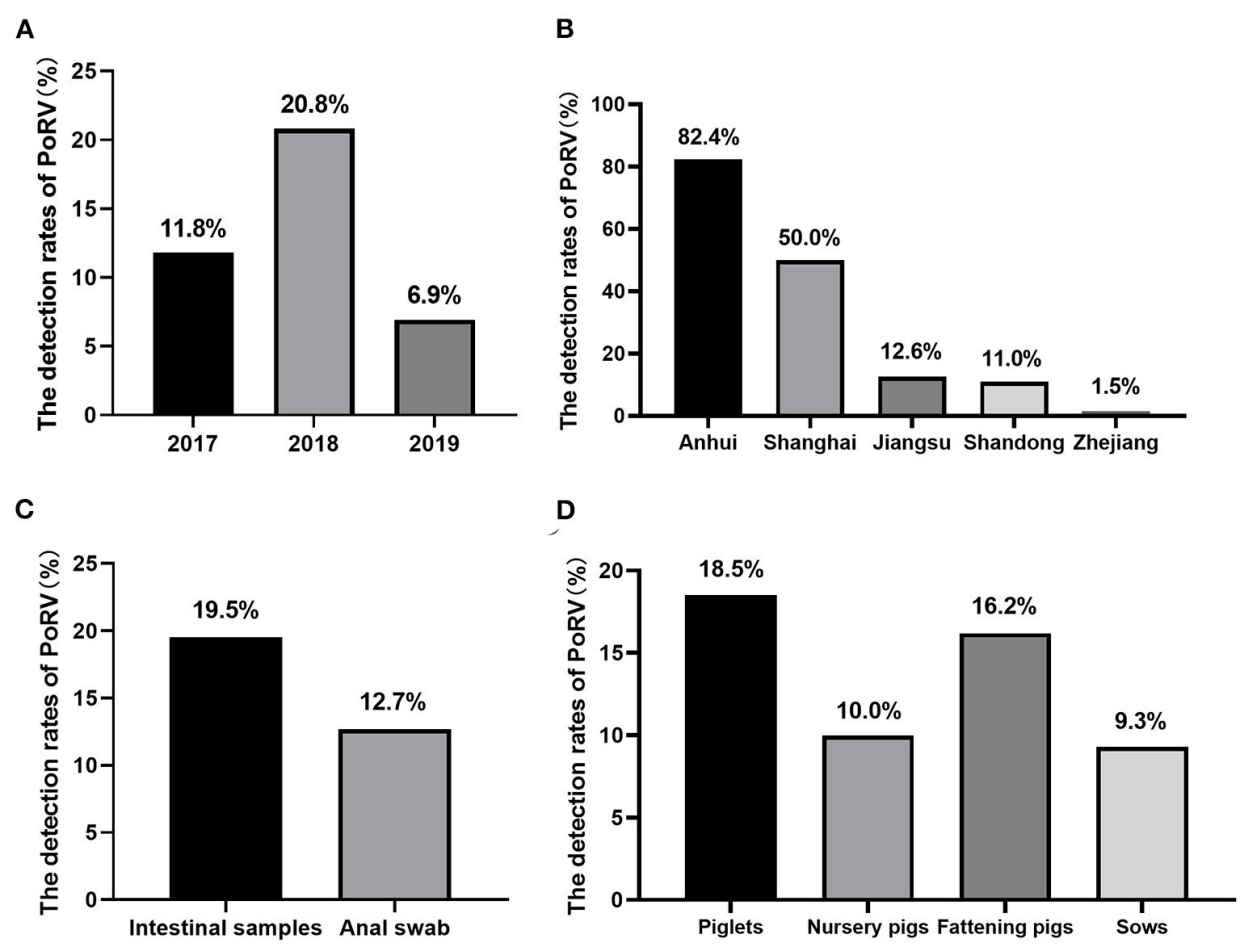
The prevalence of RVA in East China: **(A)** the positive rates of RVA of each year during this study. **(B)** The prevalence of RVA in different provinces/cities in East China. **(C)** The positive rates of different kinds of collected samples. **(D)** The detection rates of RVA in pigs at different growing stages.

A total of 594 samples were collected for detection and 100 out of 594 (16.83%) were positive for RVA by established RT-PCR. Furthermore, it was demonstrated that the positive rates of 2017, 2018, and 2019 were 11.8% (15/127), 20.8% (79/380) and 6.9% (6/87), respectively (Figure 1A). In regard to regional analysis, it was observed that the highest RVA prevalence was Anhui province (82.4%, 42/51), followed by Shanghai (50.0%, 1/2), Jiangsu (12.6%, 31/247), Shandong (11.0%, 25/228), and Zhejiang (1.5%, 1/66), respectively (Figure 1B). Besides, we also analyzed the positive rates from different kinds of samples. As shown in Figure 1C, the detection rates of intestinal samples and anal swab samples were 19.5% (68/349) and 12.7% (31/245), respectively. Also, the detection rates of RVA in relation to the age of the infected pigs were analyzed. It was demonstrated that the highest proportion was detected among piglets (18.5%, 70/379), followed by fattening pigs (16.2%, 21/130), nursery pigs (10.0%, 1/10), and sows (9.3%, 7/75), respectively (Figure 1D).

### 3.2. Phylogenetic analysis of the VP7 gene

**Figure 2:**
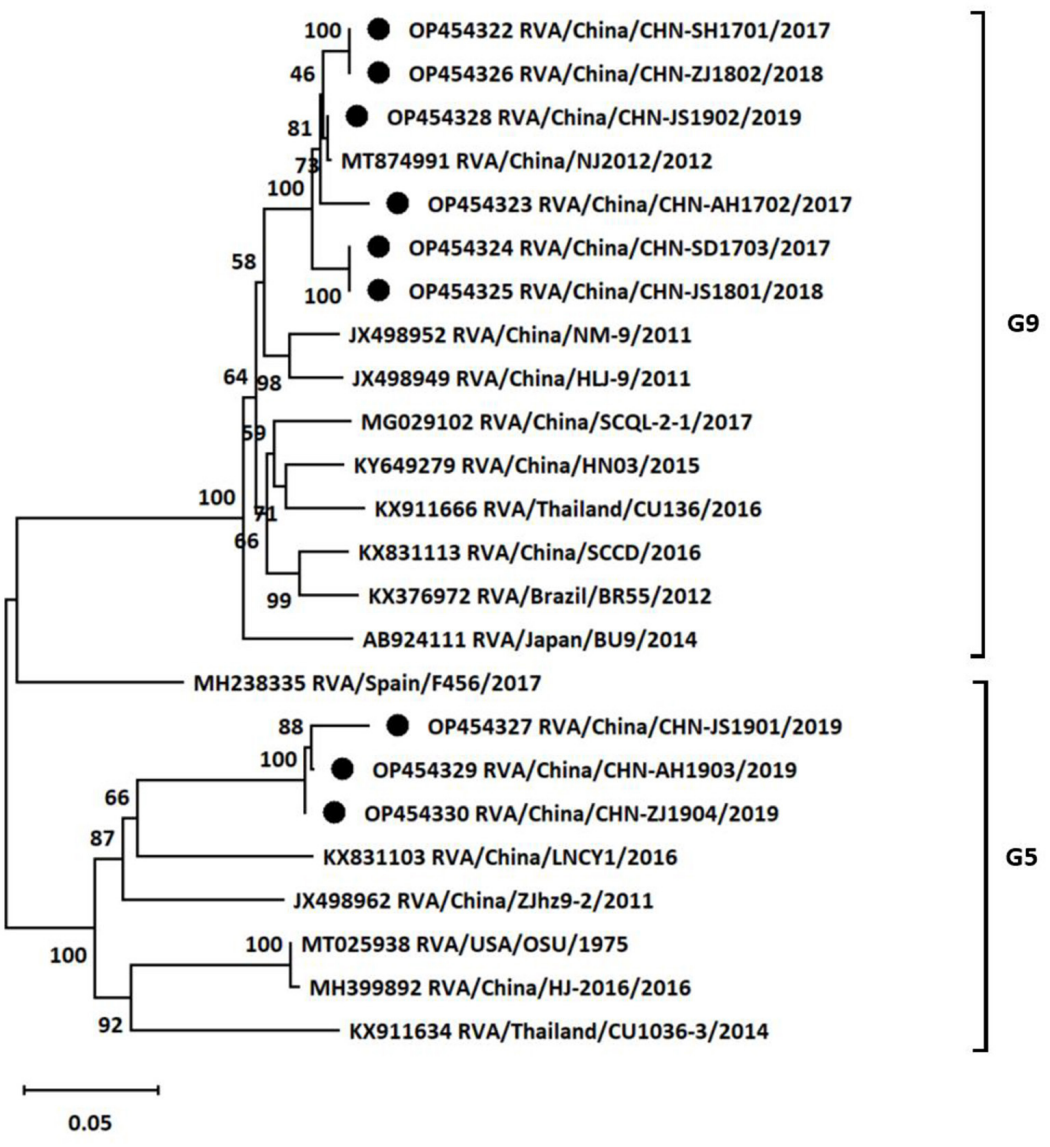
Phylogenetic tree based on the nucleotide sequences of VP7 genes from different porcine rotavirus strains: phylogenetic tree was constructed by neighbor-joining (NJ) method for the VP7 sequences of different strains using MEGA11 software. Bootstrap values were calculated on 1,000 replicates. The evolutionary distances were computed using the *p*-distance method. Other parameters were preserved by default. The nine strains from the positive samples were labeled with black circles.

To further investigate the genotypes of RVA strains circulating in East China, nine positive samples from different provinces or cities were chosen to analyze VP7 and VP4 genes by using RT-PCR. The VP7 genes of the nine different positive samples were sequenced for genotyping. Subsequently, a phylogenetic tree was established based on the VP7 gene sequences of the nine positive samples and publicly available reference strains from GenBank. Phylogenetic analysis revealed that the nine isolates from different areas and years belong to two distinct genetic groups (Figure 2). Six of nine rotavirus isolates clustered in G9 group with nucleotide *p*-distances ranging from 0.012 to 0.020 to the reference strain, while the remaining three isolates belonged to G5 group with nucleotide *p*-distances ranging from 0.127 to 0.147 to the reference strain. The VP7 sequences of CHN-SH1701, CHN-AH1702, CHN-SD1703, CHN-JS1801, CHN-ZJ1802, and CHN-JS1902 strains were most similar to NJ2012 strain (98.0%−99.9%). CHN-JS1901, CHN-AH1903, and CHN-ZJ1904 shared high homology with LNCY1 strain of 85.3%−87.3%.

### 3.3. Phylogenetic analysis of the VP4 gene

**Figure 3:**
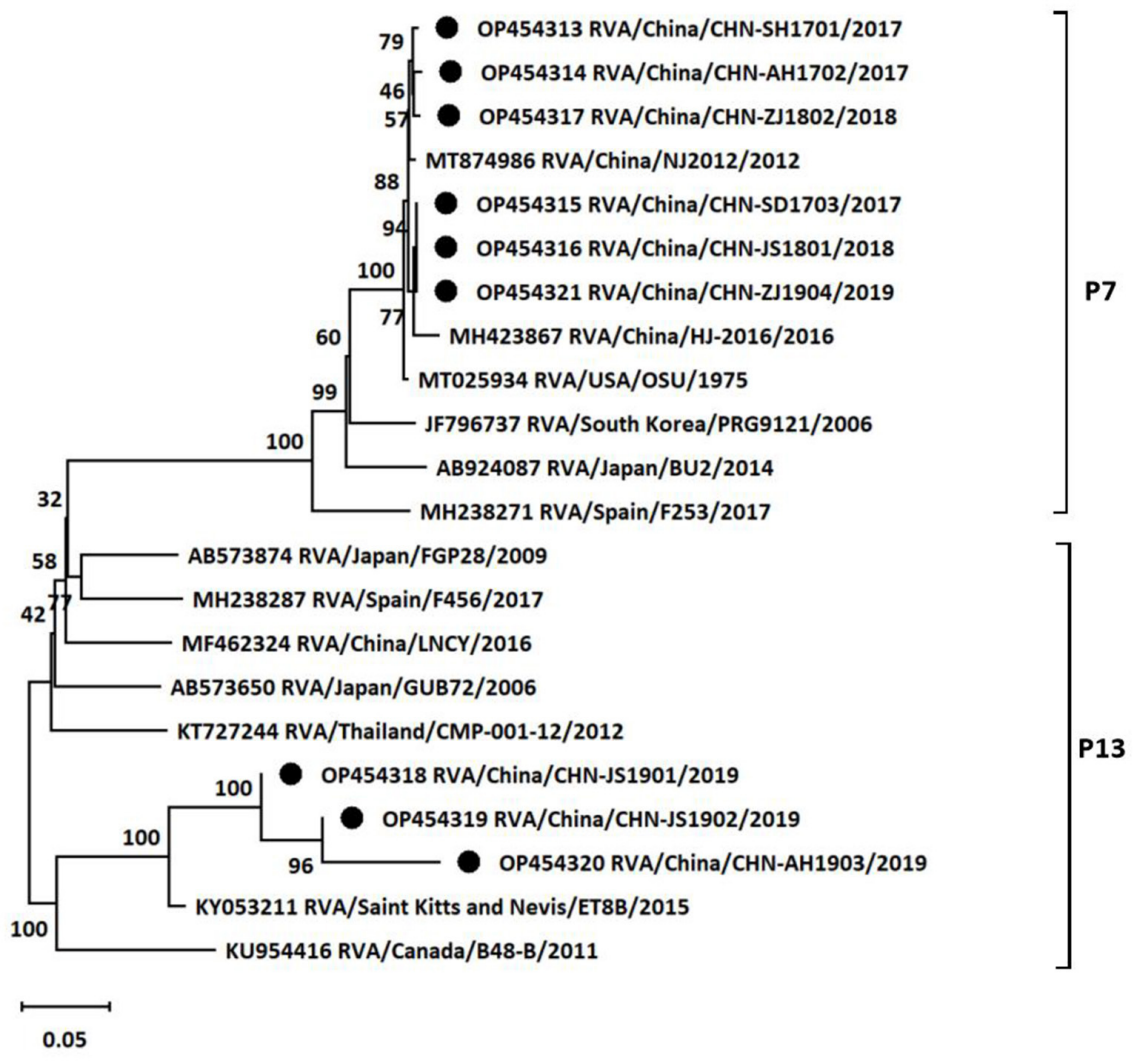
Phylogenetic tree based on the nucleotide sequences of VP4 genes from different porcine rotavirus strains: phylogenetic tree was constructed by neighbor-joining (NJ) method for the VP4 sequences of different strains using MEGA11 software. Bootstrap values were calculated on 1,000 replicates. The evolutionary distances were computed using the *p*-distance method. Other parameters were preserved by default. The nine strains from the positive samples were labeled with black circles.

At the same time, the genotypes of VP4 gene of the nine isolates were also analyzed based on sequencing and phylogenetic analysis. As shown in Figure 3, the phylogenetic tree indicated that the nine isolates belonged to two distinct genetic groups. Six of the nine rotavirus isolates were classified into P7 group with nucleotide *p*-distances ranging from 0.007 to 0.010 to the reference strain, and the rest three isolates belonged to P13 group with nucleotide *p*-distances ranging from 0.064 to 0.073 to the reference strain. CHN-SH1701, CHN-AH1702, CHN-SD1703, CHN-JS1801, CHN-ZJ1802, and CHN-ZJ1904 strains showed higher similarities (98.9%−99.3%) with NJ2012 strain. CHN-JS1901, CHN-JS1902, and CHN-AH1903 showed more closed relationship with ET8B strain (92.5%−93.5%). In summary, it was concluded that G9P[7] (five of nine) was the outstanding dominant genotype combination among the sequenced positive samples, followed by G5P[13] (two of nine), G9P[13] (one of nine) and G5P[7] (one of nine) (Table 1).

**Table 1:** Summary of genotype combinations of the sequenced samples.

**Year**	**G and P genotype**	**Number**	**Area**
2017	G9P[7]	1	Shanghai
		1	Anhui
		1	Shandong
2018	G9P[7]	1	Jiangsu
		1	Zhejiang
2019	G5P[13]	1	Jiangsu
	G5P[13]	1	Anhui
	G9P[13]	1	Jiangsu
	G5P[7]	1	Zhejiang

## 4. Discussion

Porcine diarrhea caused by viral infections continuously damages herd enteric health and leads to enormous economic loss in the global swine industry (22). However, as one of the most important pathogens causing swine acute gastroenteritis, it is the economic impact of RVA infection that cannot be ignored (3, 6, 23). Considering the impact of RVA in the swine industry, it is essential to investigate the prevalence and genetic characterization of porcine RVA in pig herds.

During 2017–2019, we investigated the presence of RVA from 35 farms collected from five provinces/cities in East China, and then carried out the phylogenetic analysis of some isolates from different areas. Our results showed that the detection rate of RVA from these farms was 16.83%. The positive rate of RVA in this study was consistent with the results presented in other studies performed in other areas in China during the same period, which suggested that PoRV seemed to be one of the most important pathogens causing diarrhea in pigs in China (24). At the same time, we also analyzed the detection rate in different years. It was noted that there was a lower detection rate (6.9%) in 2019 compared with other years. This was probably caused by the fewer samples collected from pig farms since African swine fever virus (ASFV) circulation in China. Besides, the prevalence rates of RVA in pigs varied greatly from 1.5% to 82.4% among different areas. It cannot be ignored that the impact of RVA in swine industry in Anhui province, for which the positive rate was as high as 82.4%. The samples sources were included intestinal samples and anal swab samples, and the detection rates of intestinal samples were higher than swab samples. Furthermore, RVA infections were more common in piglets and fattening pigs compared with nursery pigs and sows. These observations were also consistent with previous published study (25).

Although previous studies had investigated the prevalence rates of PoRV in several areas in China, very limited genotypes data of dominant PoRV isolates were available until now (21, 26, 27). The G/P genotype combinations of RVA identified mainly included G9P[7], G3P[13], G5P[13], G9P[13], G9P[23], G1P[7], and G9P[6] based on several isolate strains of publications during past decades in China (21, 28–33). Considering the rare data of genotypes, the VP7 gene and VP4 gene from the nine RVA strains were sequenced to demonstrate the genotypic classifications in this study. The G-types and P-types combinations in this study were also common in many areas worldwide (3). G9 and G5 types were dominant genotypes of the sequenced samples in our study, which was consistent with the previous study in Shandong province (21). Moreover, many public reports showed that positive rates of G9 and G5 types of RVA were highest in many areas of China (data not shown), so we speculated that G9 and G5 types may spread in many regions in China. However, it should be noted that there are some limitations in this study. Only nine positive samples were chosen to sequence and analyze genotypes, which could not represent the prevalent characterizations of RVA infections in the entire regions. The size of collected samples in some regions was too small to reveal the real prevalence of RVA year by year for some reasons (samples collection or samples transportations). In spite of the limitations, it was still revealed that multiple genotype combinations of RVA circulating in East China in this study. The epidemiological analysis data of RVA were several years ago in this study, which were little value for prevention the emergence of new strains. Therefore, it is necessary to maintain surveillance on the prevalent characterizations of RVA in different regions, which facilitated vaccines and other optimal strategies rational application in swine industry to prevent RVA infections.

## Data availability statement

The original contributions presented in the study are publicly available. This data can be found here: GenBank, accession numbers OP454313-OP454330.

## Ethics statement

The animal study was reviewed and approved by Jiangsu Academy of Agricultural Sciences Experimental Animal Ethics Committee.

## Author contributions

Conceptualization: BL, XZha, and XC. Methodology: XZhu, XC, and JZ. Software, data curation, writing—original draft preparation, and visualization: XC and RT. Validation: XC, KL, and LG. Formal analysis: XC, XZhu, and RT. Investigation: XC, XZhu, LG, and KL. Resources: BL, XC, KL, and XZhu. Writing—review and editing: BL, XC, RT, and SY. Supervision: BL, XZha, XC, and RT. Project administration and funding acquisition: BL. All authors have read and agreed to the published version of the manuscript.
