# Supplementary material for: Molecular epidemiological investigation of group A porcine rotavirus in East China
Source: Front Vet Sci. 2023 Mar 21;10:1138419. doi: 10.3389/fvets.2023.1138419 (PMC10070975; doi:10.3389/fvets.2023.1138419)
Supplement: Supplementary file 1 [file Data_Sheet_1.PDF]

Supplementary Material

Molecular Epidemiological Investigation of Group A Porcine Rotavirus in East China

Ran Tao[1, 2, †], Xinjian Chang[1, 2, †], Jinzhu Zhou[1, 2], Xuejiao Zhu[1, 2], Kemang Li[1, 2], Laqiang Gu[1, 2, 3], Xuehan Zhang[1, 2], Bin Li[1,2,3*]

*** Correspondence:** Corresponding Author Bin Li: Institute of Veterinary Medicine, Jiangsu Academy of Agricultural Sciences, 50 Zhong-ling Street, Nanjing 210014, China.
E-mail: libinana@126.com

1 Supplementary Tables

1.1 Supplementary Table S1. Details of sample information in this study.

Year	Farm/Area	Total samples	Herd status	Vaccination status
	Xuzhou, Jiangsu	19	Healthy sows	NO
	Xiaoshan, Zhejiang	6	Diarrhea piglets	NO
	Chuzhou, Anhui	4	Diarrhea nursery pigs	NO
	Hangzhou, Zhejiang	4	Sick piglets	NO
	Bengbu, Anhui	7	Diarrhea piglets	NO
2017	Chuzhou, Anhui	6	Sick piglets	NO
	Bengbu, Anhui	5	Diarrhea sows	NO
	Ma'anshan, Anhui	6	Diarrhea nursery pigs	NO
	Wenzhou, Zhejiang	7	Diarrhea piglets	NO
	Hangzhou, Zhejiang	8	Healthy fattening pigs	NO
	Tai'an, Shandong	19	Healthy sows	NO

	Huaian, Jiangsu	20	Diarrhea piglets	NO
	Chuzhou, Anhui	8	Diarrhea piglets	NO
	Shanghai	2	Healthy fattening pigs	NO
	Hangzhou, Zhejiang	6	Healthy sows	NO
2018	Nanjing, Jiangsu	32	Healthy piglets	NO
	Wuxi, Jiangsu	23	Diarrhea piglets	NO
	Hangzhou, Zhejiang	17	Diarrhea piglets	NO
	Linyi, Shandong	32	Healthy piglets	NO
	Linyi, Shandong	33	Healthy fattening pigs	NO
	Nanjing, Jiangsu	21	Diarrhea piglets	NO
	Tai'an, Shandong	34	Diarrhea piglets	NO
	Huaian, Jiangsu	23	Diarrhea piglets	NO
	Nanjing, Jiangsu	32	Healthy sows	NO
	Linyi, Shandong	23	Healthy fattening pigs	NO
	Tai'an, Shandong	24	Diarrhea piglets	NO
	Wuxi, Jiangsu	30	Diarrhea piglets	NO
	Linyi, Shandong	32	Diarrhea piglets	NO
	Nanjing, Jiangsu	24	Healthy piglets	NO
2019	Tai'an, Shandong	31	Healthy fattening pigs	NO
	Zhenjiang, Jiangsu	23	Healthy fattening pigs	NO

Bozhou, Anhui	5	Sick piglets	NO
Wenzhou, Zhejiang	12	Healthy piglets	NO
Chuzhou, Anhui	10	Healthy fattening pigs	NO
Hangzhou, Zhejiang	6	Sick piglets	NO

1.2 Supplementary Table S2. The primers used in this study.

Primer name	Sequences (5'-3')	Product size (bp)	Annealing Temperature (℃)
VP6-F	GGCTTTTAAACGAAGTCTTC	750	57
VP6-R	CCAGCTACYTGAATTTCTGA		
VP7-F	GGCTTTAAAAGAGAGAATTTCCGTCT	1062	55
VP7-R	TCTAAATTCTGTAGTAAAAAGCAGCTG		
VP4-F	GGCTATAAAATGGCTTCGCTCA	2362	55
VP4-R	GGTCACAACCTCTAGACACTACT		

1.3 Supplementary Table S3. Summary of RVA VP4 and VP7 genes sequenced in this study.

Virus strain	Region / GenBank accession no.	Time	Gene
CHN-SH1701	Shanghai/OP454313	2017	VP4
CHN-AH1702	Anhui/OP454314	2017	VP4
CHN-SD1703	Shandong/OP454315	2017	VP4
CHN-JS1801	Jiangsu/OP454316	2018	VP4
CHN-ZJ1802	Zhejiang/OP454317	2018	VP4
CHN-JS1901	Zhejiang/OP454318	2019	VP4
CHN-JS1902	Jiangsu/OP454319	2019	VP4
CHN-AH1903	Anhui/OP454320	2019	VP4
CHN-ZJ1904	Zhejiang/OP454321	2019	VP4
CHN-SH1701	Shanghai/OP454322	2017	VP7
CHN-AH1702	Anhui/OP454323	2017	VP7
CHN-SD1703	Shandong/OP454324	2017	VP7
CHN-JS1801	Jiangsu/OP454325	2018	VP7
CHN-ZJ1802	Zhejiang/OP454326	2018	VP7
CHN-JS1901	Jiangsu/OP454327	2019	VP7
CHN-JS1902	Jiangsu/OP454328	2019	VP7
CHN-AH1903	Anhui/OP454329	2019	VP7
CHN-ZJ1904	Zhejiang/OP454330	2019	VP7

1.4 Supplementary Table S4. PoRV reference strains associated with VP7 gene described in this study.

Virus strain	Countries / GenBank accession no.	Time	G type
OSU	USA/MT025938	1975	G5
HJ-2016	China/MH399892	2016	G5
LNCY1	China/KX831103	2016	G5
ZJhz9-2	China/JX498962	2011	G5
CU1036-3	Thailand/KX911634	2014	G5
CMP-138-14	Thailand/KT727263	2014	G5
F456	Spain/MH238335	2017	G5
NM-9	China/JX498952	2011	G9
HLJ-9	China/JX498949	2011	G9
HN03	China/KY649279	2015	G9
SCCD	China/KX831113	2016	G9
SCQL-2-1	China/MG029102	2017	G9
CU136	Thailand/KX911666	2016	G9
BR55	Brazil/KX376972	2012	G9
RV0146	USA/KC244287	2012	G9
BU9	Japan/AB924111	2014	G9

1.5 Supplementary Table S5. PoRV reference strains associated with VP4 gene described in this study.

Virus strain	Countries / GenBank accession no.	Time	P type
OSU	USA/MT025934	1975	P[7]
PRG9121	South Korea/JF796737	2006	P[7]
BU2	Japan/AB924087	2014	P[7]
HJ-2016	China/MH423867	2016	P[7]
NJ2012	China/MT874986	2012	P[7]
F253	Spain/MH238271	2017	P[7]
B48-B	Canada/KU954416	2011	P[13]
FGP28	Japan/AB573874	2014	P[13]
LNCY	China/MF462324	2016	P[13]
GUB72	Japan/AB573650	2006	P[13]
F456	Spain/MH238287	2017	P[13]
ET8B	Saint Kitts and Nevis/KY053211	2015	P[13]
CMP-001-12	Thailand/KT727244	2012	P[13]
